# Sustaining youth physical activity in times of challenge and change: lessons from COVID-19

**DOI:** 10.1093/heapro/daad038

**Published:** 2023-05-04

**Authors:** Suzanne Trask, Peg Lockyer, Jillian Hildreth, Erica D’Souza, Tatjana Buklijas, Rochelle Menzies, Mark Vickers, Jacquie L Bay

**Affiliations:** Liggins Institute, University of Auckland, Park Road, Grafton, Auckland, New Zealand; Liggins Institute, University of Auckland, Park Road, Grafton, Auckland, New Zealand; Liggins Institute, University of Auckland, Park Road, Grafton, Auckland, New Zealand; Liggins Institute, University of Auckland, Park Road, Grafton, Auckland, New Zealand; Koi Tū Centre for Informed Futures, University of Auckland; Koi Tū Centre for Informed Futures, University of Auckland; Liggins Institute, University of Auckland, Park Road, Grafton, Auckland, New Zealand; Liggins Institute, University of Auckland, Park Road, Grafton, Auckland, New Zealand; Koi Tū Centre for Informed Futures, University of Auckland

**Keywords:** sustaining physical activity behaviours, youth physical activity, COM-B, COVID-19, life stage transitions, resilience, physical activity enablers and barriers

## Abstract

Physical activity (PA) is recognized as essential for positive physical and mental well-being in young people. However, participation in PA is known to decline as adolescents emerge into adulthood under the influence of complex social and structural factors. Globally, COVID-19 restrictions resulted in changes to PA and PA participation levels in youth populations, providing a unique opportunity for gaining insight into PA barriers and enablers in circumstances of challenge, limitation and change. This article details young people’s self-reported PA behaviours during the 4-week 2020 COVID-19 lockdown in New Zealand. Taking a strengths-based view and drawing on the COM-B (capabilities, opportunity and motivation behaviour) model for behaviour change, the study explores factors enabling young people to sustain or increase PA during lockdown. Findings are drawn from qualitative-dominant mixed-methods analyses of responses to an online questionnaire: New Zealand Youth Voices Matter (16–24 years; *N* = 2014). Key insights included the importance of habit and routine, time and flexibility, social connections, incidental exercise and awareness of links between PA and well-being. Of note were the positive attitudes, creativity and resiliency demonstrated as young people substituted or invented alternatives to their usual PA. PA needs to change to adapt to new circumstances over the life course, and youth understanding and knowledge of modifiable factors may provide support for this. Thus these findings have implications for sustaining PA during late adolescence and emerging adulthood, a life phase that can be associated with significant challenge and change.

## INTRODUCTION

Higher levels of physical activity (PA) are associated with positive mental and emotional well-being and lowered risk of developing non-communicable diseases in youth aged 16–24 years (van Sluijs *et al.*, 2021). Therefore, increasing participation in regular and recommended levels of PA in this group is a priority. Concerningly, there is evidence that in New Zealand and internationally, levels of PA sufficient for maintaining positive overall well-being are not met, as PA tends to decline between childhood and adolescence, and again in the transition from high school to university or employment (Corder *et al.*, 2015; Van Dyck *et al.*, 2015; Gray *et al.*, 2019; Gropper *et al.*, 2020; Kemp *et al.*, 2020; O’Connor and Penney, 2021). Complex personal, sociocultural and socioecological factors influence PA in young people and these factors need to be understood when designing intervention supports. For example, individual PA competencies, preferences, attitudes and motivations are influenced by social interactions with family and peer groups, and by cultural and societal values and beliefs. Environmental settings such as home and lifestyle, the wider community and built environment, and the nature of involvement in education or work also influence opportunities available for PA (Deliens *et al.*, 2015; Martins *et al.*, 2015; Rhodes and Rebar, 2017). In the built environment, active transportation depends on distance and opportunities for safe passage. Also significant, late adolescence and emerging adulthood are times of transition and change to living, working or studying independently. Additional factors such as choices to prioritize study or socializing, distancing from parental preferences and family support, changes in leisure time, and challenges such as limited financial means are introduced (Van Dyck *et al.*, 2015; Gropper *et al.*, 2020; Mascherini *et al.*, 2021; Teare and Taks, 2021). Understanding why and how young people might sustain PA participation in circumstances of challenge or change is therefore important in promoting and increasing PA engagement.

The purpose of this article is to examine factors that enabled young people aged 16–24 years to sustain or increase PA during the 4-week COVID-19 lockdown in New Zealand in 2020, and where PA decreased, to understand barriers that contributed to this. During the lockdown, people were instructed to stay at their place of residence. No gatherings were permitted, and all public and education facilities were closed. Only essential businesses and services operated. Exercise was permitted only within living confines and proximal neighbourhood areas. Any activities that might have resulted in injury requiring hospital care or rescue services were not permitted (Ministry of Health, 2021). These exceptional restrictions provide a unique opportunity for gaining insight into PA barriers and enablers for youth in conditions of change, challenge and limitation.

The term PA refers to any behaviour that involves sustained movement, including organized, non-organized and incidental PA. Exercise can be considered a subcategory of PA, delineating planned, structured or repetitive activity associated with the intent to maintain physical fitness (World Health Organisation, n.d.).

### Impacts of COVID-19 on levels of PA

The pandemic has influenced PA for people of all ages worldwide (Tison *et al.*, 2020). Teare and Taks (Teare and Taks, 2021) hypothesized that preferences for non-organized PA would increase during the pandemic and reasons for PA participation would shift towards prioritizing well-being. A survey by Mascherini *et al*. (Mascherini *et al.*, 2021) of staff and students at the University of Florence, Italy (*N* = 1383) demonstrated that work-related PA declined but recreational PA increased. Constandt *et al*. (Constandt *et al.*, 2020) found that PA in adults aged 18 and over was influenced by whether or not exercise was conducted as part of a social group and whether online tools were accessed to support PA. Adults who were usually sedentary prior to lockdowns self-reported higher levels of PA during the COVID-19 lockdowns, however, adults who were active in sports groups were less likely to exercise when deprived of their usual form of PA. These findings direct attention to the complexity of interconnected drivers of PA and have relevance for young people who were likely to miss involvement in organized sports but more likely to engage in high levels of screen time and sedentary behaviours during the pandemic (Medrano *et al.*, 2021).

In many countries, the COVID-29 pandemic led to the closure of sports facilities and parks, postponed events and team sports, and tight restrictions on movement. Opportunities for young people to engage in PA were severely curtailed, and PA was carried out indoors and/or close to home (Chen *et al.*, 2020; Dunton *et al.*, 2020; Xiang *et al.*, 2020; Medrano *et al.*, 2021; Stockwell *et al.*, 2021; Alcántara-Porcuna *et al.*, 2022). Dunton *et al*. (Dunton *et al.*, 2020) identify that COVID-19 restrictions made it difficult for children and young people to achieve recommended levels of PA. Reporting from the USA, they caution that decreased levels of PA may become entrenched and in the short term recommend programmatic and policy strategies to reverse this trend. Other research has similarly highlighted the urgency of supporting a return to healthy recommended levels of PA. In Germany, representative survey data from a study by Mutz (Mutz, 2021) (≥14 years, *N* = 1001) showed that a reduction in PA was associated with a decline in emotional well-being during the pandemic, however, individuals who stayed physically active were most able to maintain positive or pre-pandemic levels of well-being.

Reporting from China, Chen *et al*. (Chen *et al.*, 2020) urge parents and education communities to remain mindful of the long-term impacts on children of decreased PA associated with increased risks of developing chronic disease in later life. They suggest that a return to normality as schools and tertiary institutes remain open presents opportunities to establish new strategies for promoting PA-related behaviours in young people.

Some have argued that declining levels of PA during the pandemic were expected with, for example, less work-related PA or active travel to and from the place of work or education. To date, surveys of PA during COVID-19 have recognized changes and general decline (Mascherini *et al.*, 2021). Other research suggests that the pandemic necessitated the finding of innovative ways to inspire continued participation in PA (Hayes, 2022). Commenting on patterns in PA, Eime *et al*. (Eime *et al.*, 2022) suggest that COVID-19 restrictions negatively impacted PA for individuals who already led a primarily sedentary lifestyle but that the pandemic supported some individuals to prioritize PA and begin a more active life. Constandt *et al*. (Constandt et al., 2020) noted this positive trend. There has been limited commentary around strategies that might work to inform, educate and support youth to sustain PA during times of significant challenge or change. If it is possible to sustain or increase levels of PA in restricted circumstances such as a pandemic-related lockdown, this has positive implications for future challenges, and it would seem important to learn from this.

### The COM-B model (capabilities, opportunity and motivation behaviour)

Young people vary widely in their capabilities, opportunities and motivations around PA, and literature in this field is concerned with identifying the complex and qualitatively different determinants of PA behaviours (Martins *et al.*, 2015; Corr *et al.*, 2019). Sustaining PA over the long term can require high levels of intrinsic or autonomous motivation and demand a degree of effort, reinforcing the need for external structures and supports associated with, for example, organized PA at schools and sports clubs (Teixeira *et al.*, 2012). Informed by self-determination theory, Dishman *et al*. (Dishman *et al.*, 2018) point out that older adolescents and adults’ PA choices are dependent on intrinsic motivation and personal values to the point where ‘being’ a physically active person may (or may not) become part of their core identity. Intrinsic motivations include those related to participation for its own sake, enjoyment, health benefits, body condition, challenge, fitness or skill development, or personal growth. These can be distinguished from extrinsic motivations related to reward, recognition by others or avoidance of negative consequences (Teixeira *et al.*, 2012).

Determinants of PA incorporate intrinsic and extrinsic motivations and external structures that enable or hinder PA engagement. In 2011, Michie, van Stralen and West pointed to the centrality and value of the COM-B model (capabilities, opportunity, motivation and behaviour), used to support the design of PA behavioural interventions. The COM-B constructs have been widely used, for example, to identify barriers and enablers to PA in school settings and for adolescents with intellectual disabilities (Howlett *et al.*, 2019; Rosenkranz *et al.*, 2021; McDermott *et al.*, 2022). The model provides a conceptual framework for identifying complex and interconnected modifiable and non-modifiable behavioural determinants of PA, including cognitive, affective, social and environmental influences. Capability refers to an individual’s physical skills together with psychological ability including knowledge supporting them to carry out a behaviour. The construct of motivation emphasizes goals that drive the behaviour along with the thought processes, beliefs and socioemotional responses that strengthen and guide behaviour. Opportunity draws attention to external environmental and structural factors as well as social, cultural and financial factors beyond the individual that can influence the enablement of the behaviour. According to the COM-B model capability, opportunity and motivation must be sufficient for a PA-related behaviour to occur, however, each of the constructs has an influence on the other. For example, capability and opportunity can together or separately influence motivation. Equally, motivation and opportunity together can contribute to improved capability, both in the sense of improved competence or skill and in the ability to initiate and sustain participation (Michie *et al.*, 2011).

In this study the COM-B constructs provide a framework for a strengths-based analysis of influences on PA behaviour in young people, accounting for a complex interplay of self-efficacy and affordances. Below we explain the consequence and relevance of the strengths-based perspective.

### A strengths-based view

The pandemic has heightened interest in how humans adapt to challenging or uncertain circumstances to preserve a state of positive overall well-being. Armstrong *et al*. (Armstrong *et al.*, 2020) argue that strengths-based approaches are of value as communities seek to move on from exigent pandemic challenges to an endemic recovery phase requiring resilience and adaptation across many aspects of daily life. Positive psychologists Lopez and Gallagher recommend that, ‘using the same techniques and tools that help us explain weakness and prevent or treat illness, we could enhance our ­understanding of strengths and promote well-being’ (2009, p. 3).

Strengths-based approaches are recognized and well-documented ways of generating insight into what works and what might be possible across a range of research with salutogenic aims (McCuaig and Hay, 2013; Enright *et al.*, 2014; Clouder *et al.*, 2016; Gray *et al.*, 2019; Quennerstedt, 2019; Sargent and Casey, 2021). Internationally, strengths-based approaches provide an orientation to well-being inquiries in Health and Physical Education curricula (McCuiag et al., 2013). Seeking to enhance engagement in school-based physical education, Gray *et al*.’s (2019) research underscores the value of understanding and sharing stories of success from students themselves. Strengths-based approaches involve the systematic search for those aspects of organizations, communities, events, practices or individual qualities that are most positive or effective. This knowledge is used to imagine ideal or preferred outcomes and to decide on, plan for and execute solutions to challenges (Cooperrider and Fry, 2020; Sargent and Casey, 2021).

Strengths-based approaches are not about relentless and naïve positivity but are an orientation towards resilience and seeking possibilities, no matter how challenging the circumstances. As Dvorsky *et al*. (Dvorsky *et al.*, 2021) point out, a sole focus on difficulties carries a risk of ignoring what is positive, and problems can grow to feel overwhelming. In addition, negative or deficit approaches can position those coping with the issue as helpless or unable to change. Instead, it can be beneficial to look ‘beyond what is broken’ (Enright *et al.*, 2014, p. 912), viewing negative aspects as a springboard for change.

## RESEARCH CONTEXT

### The New Zealand 2020 COVID-19 lockdown

The New Zealand Government implemented a ‘go hard, go early’ approach in March 2020 when evidence of COVID-19 community transmission first appeared (Baker *et al.*, 2020). A four-tier alert level system defined restrictive measures with Level 4 representing the greatest risk (New Zealand Government., 2022). The 4-week Level 4 lockdown began March 25 and ended 27 April 2020. Travel was restricted, and all non-essential services including gyms and sporting facilities were closed. Universities, schools and other education providers were shut, and learning moved online. This resulted in the closure of schools and universities and the mass mobilization of student populations back to their family residences. Exercise was permitted only within living confines and proximal neighbourhood areas. Any activities that might have resulted in injury requiring hospital care or rescue services were not permitted; for example, swimming, surfing, mountain biking, hiking, hunting, fishing and boating.

## RESEARCH METHODS

The question guiding this research is: what can we learn from young people’s (16–24 years) self-reported habits and behaviours around PA during the 4-week COVID-19 Level 4 lockdown in New Zealand that might support them to sustain healthy levels of PA during times of challenge or change?

Rapid online surveys are an effective tool for understanding how population groups experienced pandemic-related lockdowns (Geldsetzer, 2020). Data are drawn from questionnaire items related to self-reported levels of participation in PA (youth 16–24 years) during the 4-week COVID-19 2020 lockdown. This anonymous online New Zealand COVID-19 Youth Voices Matter questionnaire was administered by transdisciplinary researchers in response to the need to understand the impacts of the pandemic on young people. The questionnaire targeted 16- to 24-year-olds living in New Zealand during the period March to June 2020. It was conducted in late May and throughout June of 2020, 1–2 months after the end of Level 4 lockdown. At the time, New Zealand was under Level 2 restrictions. Schools, universities, gyms and sports facilities were open, subject to meeting health requirements. Likert and open-ended questions focussed on: basic knowledge relating to COVID-19, the New Zealand Government’s COVID-19 response, COVID-19 information sources used, impacts of social restrictions on learning and/or work, nutrition and PA. The questionnaire included items on age, education and other sociodemographic factors (e.g. housing/accommodation). A social media recruitment strategy was used to attract participants representing diverse youth populations. Social media advertisements targeted required demographics to support representative sampling for gender, ethnicity and geographical areas.

Data were collected from 2048 qualifying respondents between the ages of 16 and 24 years. Respondents that did not enter any fields past Question 3 (*n* = 34) were excluded from the data set, resulting in a reduced data set of 2014. Consent was obtained prior to the start of the questionnaire; the recruitment page included a link to the participant information sheet. Ethical approval was obtained from the University of Auckland Human Participants Ethics Committee (UAHPEC) (Reference number 24,677).

Closed-answer questions elicited information on whether and how levels of PA changed during lockdown. First, respondents were asked: ‘During Alert Level 4 lockdown, did the amount of PA you took part in: Increase/decrease/stay the same?’ Respondents were then asked, ‘During lockdown, did you change the way you exercised?’ If the answer was Yes, an open-text response item invited participants to explain, ‘Please tell us how?’

### Data analysis

The focus in this paper is on understanding factors that enabled young people to maintain or increase levels of PA during the lockdown, and where PA decreased, to understand barriers that contributed to this. A qualitative-dominant mixed-methods approach was applied in the analysis.

Chi-squared tests of independence were used to assess the significance of the association between categories: gender, education/work status and age. If results indicated a significant association, *post hoc* analyses with adjusted standardized residuals were conducted to test for higher or lower-than-expected frequencies.

Qualitative inductive thematic analysis was conducted of open-text responses where participants described how they changed the way they exercised. This analysis generated categories describing self-reported motives, supports or inhibitors related to increased or decreased levels of PA, and whether and how the mode of PA changed. The analysis was conducted via a team manual coding process using NVivo 12Pro. Cohen’s Kappa coefficient was used as a measure of inter-coder reliability (ICR). The intent in calculating ICR was to support high standards through external accountability as coders worked through large data sets, and to enhance coding consistency by promoting discussion and dialogue within pairs (Braun and Clarke, 2013; O’Connor and Joffe, 2020). Using iterative analyses an average overall agreement of 0.75 was expected. Overall themes were derived from clustering categories and sub-categories. Descriptive analyses in the form of percentage values from clustered open-ended response categories are included in findings to give an indication of typicality or relative importance of an issue, contributing to the internal generalizability of the collection of responses (Maxwell, 2010). In subsequent deductive analyses a strengths-based framework based on broad constructs from the COM-B model provided further insight into important drivers and influences on PA during the lockdown.

## FINDINGS

Table 1 details percentage changes in PA participation by gender, ethnicity and education/work status.

**Table 1:** Respondent demographics and PA participation

Gender	New Zealand Pākehā/European	Māori	Pacific (Samoan, Cook Islands Māori, Tongan, Niuean)	Chinese	Indian	Other	Prefer not to say	Total (respondents could select more than one category)	Increased PA	Decreased PA	PA unchanged
Male	72.9%	12.2%	8.5%	7.2%	3.2%	15.1%	1.7%	120.8%	31%	47%	22%
Female	79.6%	14.0%	1.8%	4.6%	3.3%	12.6%	1.5%	119.6%	35%	45%	20%
Gender diverse	88.9%	20.0%	0.0%	0.0%	2.2%	4.4%	0.0%	115.6%	18%	60%	22%
Prefer not to say	52.9%	5.9%	0.0%	0.0%	5.9%	0.0%	29.4%	100.0%	35%	41%	24%
** ** **Change in PA levels**											
Increased PA	32%	33%	34%	28%	38%	37%	7.14%	—	—	—	—
Decreased PA	47%	41%	36%	53%	50%	44%	60.71%	—	—	—	—
PA unchanged	21%	26%	30%	19%	12%	18%	32.14%	—	—	—	—
** ** **Education/work status**											
Secondary school student	—	—	—	—	—	—	—	—	38%	42%	20%
Tertiary student	—	—	—	—	—	—	—	—	27%	55%	19%
Employed	—	—	—	—	—	—	—	—	32%	45%	23%
Unemployed	—	—	—	—	—	—	—	—	23%	47%	29%
Apprenticeship	—	—	—	—	—	—	—	—	25%	46%	29%

A chi-squared test of independence showed no significant association between self-reported changes in PA levels and male and female groups X2 (3, *N* = 1553) = 2.9, *p* = 0.236699. Gender-diverse people were more likely to report decreased PA. (‘Unspecified’ category was left out because expected cell count <5.)

There was a significant relationship between study/employment status and change in PA, X2 (8, *N* = 1576) = 33.2, *p* = 0.000058. *Post hoc* analysis with adjusted standardized residuals showed that high school students were significantly more likely to report decreased PA and significantly less likely to report increased PA. The reverse was true for tertiary students, who were significantly more likely to report increased PA and significantly less likely to report decreased PA.

There was a significant relationship between age and change in PA, X2 (16, *N* = 1576) = 32.4, *p* = 0.00877034. *Post hoc* analysis with adjusted standardized residuals showed that people aged 17 years were more likely to report increased PA and less likely to report decreased PA.

Working out at home, walking and running were the most common forms of exercise for those who increased PA during lockdown. Other PA included using rowing machines at home, dancing, skating or playing basketball. One individual described long walks down nearby train tracks as there were no trains running. Cycling was popular as a new activity or substitution for usual exercise PA. Three young people pointed out that they felt safe to bike on empty roads during lockdown, whereas they normally did not:

I never normally bike ride because I don’t like biking around a lot of cars. [Female, 16 years, school student]

Having time to exercise was mentioned as a key factor in increased activity. A typical response was,

I finally had time to exercise [Male, 17 years, school student]

Having more time was related to the establishment of a daily routine for 13% of respondents in this category, as illustrated by the participant who noted that,

I had more time to run, so I did most days. [Female, 17 years, school student]

Having too much time was an issue for some, and an exercise routine helped to structure the day. In the words of one young person:

Cause what else was there to do? It helped create a routine. [Female, 20 years, tertiary student]

As options for PA were limited, creative substitution of one form of PA for another supported many young people to increase PA:

I set up a gym in the garage with what we had lying around. [Male, 16 years, school student]

For informants in this category, important enablers for higher levels of PA included more time and flexibility; greater opportunity to engage in PA; social connections including friends and family; creative substitution; and motivations based on a desire to maintain fitness, get outside or provide variety.

While a small number struggled with motivation without social support, the most significant proportion of young people mentioned the loss of their usual space, place or structures supporting exercise as a factor that negatively their impacted levels of PA. For this group, substitution led to an overall decrease in intensity or duration of PA, as exemplified by the response:

I usually go to the gym, went on daily walks instead which is a less intense workout. [Female, 24 years, employed]

Those who relied on incidental exercise such as active travel to school, university or work, or work-related PA, did less or no PA at all when this option disappeared. There were many examples, including:

I was not doing as much exercise as I was not walking to friends’ houses and home from school [Female, 16 years, school student]

I do a lot of walking at uni, so that decreased [Male, 22 years, tertiary student]

I wasn’t biking to work anymore. [Female, 23 years, employed]

I stopped my gardening apprenticeship [Male, 23 years, apprentice]

Those living in rural areas on a 100 km/h rural road were less likely to exercise by walking, and those living with disabilities were limited in the type of PA were able to engage in. A small number of respondents were living with injury or mental health issues that impacted PA, while for some the fear of contracting COVID-19 influenced their movement outside of their place of residence. One young person commented,

I spent hours walking laps around my small apartment, too scared to go outside. [Gender diverse, 23 years, employed]

This data highlights complex factors impacting young people’s opportunities and ability to participate in a variety of forms of PA during lockdown. Often these were interconnected. Drawing together two elements of active transport as incidental exercise and the lack of social contact, one young person stated that,

My exercise is everyday activity, walking, usually in between or during social gatherings. I struggle to exercise alone, purely for the purpose of exercising. [Female, 2 years, unemployed]

Another example of the interconnected nature of influences was the respondent who commented that,

I used to be going out and visiting people or going on a walk. I lacked energy and motivation to do anything, as well as not having a job where I was using my body or standing all day. [Female, 21 years, employed]

Of those who maintained similar levels of PA during lockdown, the majority pointed to the substitution of one form of PA for a permitted or possible mode, with walking becoming the primary mode. One respondent reported that they went on walks enjoy nature rather than using walking as a form of transport [Female, 18 years, tertiary student]. Other typical responses included:

Biking instead of the gym and football. [Male, 16 years, school student]

I spent more time walking outside since the complex gym was closed, and my normal running partner was not in my bubble. [Male, 23 years, employed]

### COM-B constructs

Six broad strengths-based themes are presented in Table 2. Themes are illustrated using a sample of reported behaviours (indicative quotes) across the COM-B constructs. However, as discussed, it is often difficult to isolate COM-B influences (Howlett *et al.*, 2019). The data in Table 2 suggests that at least two constructs were in play and visible in all situations of reported behaviour. For instance, a young person making an effort to go on a daily walk might draw attention to their knowledge of health benefits (capability) that fed into their increased motivation to undertake the behaviour. The third construct (opportunity) might be assumed to be influencing behaviour, for example, having increased time for PA; but not be visible in the data. The following quote indicates that one young person recognized their reliance on social interactions (motivation) and exercise facilities (opportunity). During the lockdown removal of these led to PA substitution (capability) requiring effort and intentionality; in other words, they needed to be more deliberate in finding opportunities due to lower motivation:

**Table 2:** Indicative quotes: COM-B constructs related to increased PA

Strengths-based theme	COM -B constructs visible in reported behaviour	Illustrative quotes from respondents
The value of routine or habit	Motivation to begin PA leads to development of capability and sustaining PA	*I actually started to exercise and had a weekly routine* [Male, 17 years, school student] *I started going on walks at the start and then that escalated to running and now I do cardio too* [Female, 17 years, tertiary student] *I started a habit of daily one hour bike rides and started learning yoga* [Female, 20 years, tertiary student]
	Where a PA routine was previously important, capability and motivation to maintain this	*I had to deliberately go out to exercise because it was no longer part of my routine* [Female, 22 years, tertiary student] *My exercise routine before lockdown consisted of a biking an average of 30kms a day. Plus exercises with weights such as squatting. However when lockdown began I changed my program to running about 7kms a day with full body workouts* [Male, 17 years, school student]
	Where a PA routine was previously important, motivation to sustain and capability to substitute	*I began replacing some of my older exercise habits with other things, for example working out in the garage instead of doing P.E. at school* [Male, 17 years, school student] *I am a swimmer and lockdown prevented me from training in a pool. This meant I had to change all of my training to be land based or ocean swimming (when allowed)* [Female, 17 years, school student]
The influence of increased time and flexibility	Increased time and flexibility mean increased opportunity, motivation to use the time for PA	*I went on lots of long walks and bike rides, something I don’t usually have time for* [Female, 18 years, employed] *I began doing push-ups, sit-ups and squats in the morning and evening, since I had more free time.* [Male, 16 years, school student] *I have more time to exercise and was more flexible with the time of day* [Female, 16 years, school student] *I had more time because I didn’t have after school activities or long car rides* [Female, 16 years, school student]
The power of initiative and creative substitution	Lack of usual opportunity but motivation and capabilities to find new alternatives and thus new opportunities	*I couldn’t play team sports or go to the gym, so I got creative (used rice bags as weights, etc.)* [Male, 16 years, school student] *I did a lot more workouts inside and used furniture as makeshift gym equipment* [Male, 17 years, school student] *Instead of strength training at the gym we had to modify it for home and added some yoga* [Female, 22 years, employed] *I went from depending on organised activities such as football training to requiring the initiative to exercise such as running* [Male, 17 years, school student]
The benefits of social connections	Motivation was increased by opportunities created by social interaction	*I would go on runs if someone else went with me [Male, 16 years, school student]* *My friends and I would zoom call each other every day to do a half an hour YouTube workout* [Female, 16 years, school student] *Started doing exercises as a flat so we would motivate each other to keep going.* [Female, 21 years, tertiary student] *My sister was with us and she always wanted me to workout with her* [Female, 18 years, tertiary student]
Awareness of the link between PA and well-being	Knowledge of the importance of health or fitness (capability) increased motivation to increase or sustain PA	*I took up running and started looking at other ways to keep fit and build muscle* [Female, 16 years, school student] *I made sure I did home workouts every day to improve my mental health* [Female, 20 years, unemployed] *I started going out for runs which helped me stay healthy in the tough situation we were all in* [Male, 16 years, school student]
The importance attached to incidental exercise	Lack of usual opportunity but motivation to continue with substitute PA	*Normally I would get a lot of exercise going about my day but as we had to stay home I had to convince myself to actually exercise rather than doing it subconsciously. I would have to go for walks* [Female, 21 years, employed] *My work is physical, I couldn’t do that in lockdown, so I walked (a lot!) and did body weight exercises instead* [Female, 17 years, school student] *I had to make the effort to go out for a walk rather than being able to have a high number of steps just by walking around places all day* [Female, 16 years, school student]

I had to look to YouTube workouts and walks with the dogs to substitute the strength training I usually do at the gym. It was a lot harder as I didn’t have the equipment or motivation without the social aspect. [Female, 23 years, employed]

## DISCUSSION

Globally, COVID-19 restrictions resulted in changes to PA and participation levels in youth populations, providing a unique opportunity for gaining insight into PA barriers and enablers in circumstances of challenge, limitation and change. This research investigated young people’s self-reported PA behaviours during the 4-week 2020 COVID-19 lockdown in New Zealand. Taking a strengths-based view and drawing on the COM-B (capabilities, opportunity and motivation behaviour) model for behaviour change, this research explored factors enabling young people to sustain or increase PA during lockdown, and where PA decreased, explored barriers that contributed to this.

The study found that tertiary students were more likely to report increased PA during lockdown when on-campus accommodations had closed and students returned to their usual places of residence, while secondary school students were more likely to report a decrease in PA during lockdown. These findings are interesting given the trend of declining PA as young people transition from high school to tertiary studies or employment (Corder, 2015; Van Dyck *et al.*, 2015; Gropper *et al.*, 2020). This suggests that for some young people, the habits and affordances associated with a home environment, once removed, are not easily replaced. Outside of the lockdown situation, it is possible that transitions away from home and the removal of structures and supports associated with home and school environments (e.g. school-based physical education programmes, organized school- or community-based fixtures and PA opportunities, parental encouragement, parental provision of transport or finance for PA pursuits), negatively influences PA. Further research is required to understand these findings.

Significant for this research were the young people who demonstrated were able to harness motivations and capabilities and find or create ways to stay physically active during lockdown. This group tended to take a problem-solving approach. They adapted, balanced demands on their time and looked for opportunities to engage regularly in PA (Teixeira *et al.*, 2012). They managed this by accessing online social support which is known to be an important motivator (Constandt *et al.*, 2020), making their own equipment, or designing their own workout programmes. This finding aligns with Teare and Taks (Teare and Taks, 2021) suggestion that COVID-19 limitations would expose youth to new and creative ways of participating in sport and physical activities, thus shaping new preferences.

It is possible that past preferences and experiences of regular, satisfying, competitive or enjoyable PA contributed to the development of capabilities that positively impacted motivation levels for this group. Consistent with this, Mascherini *et al*. (Mascherini *et al.*, 2021) suggest that PA is an intentional activity, influenced by self-efficacy that determines how people think, behave and feel. A high level of self-efficacy was exemplified by respondents who stated they ‘had to’ make an effort or convince themselves to exercise when they were not inclined, or ‘had to’ substitute one form of PA for another. An indication of the strength of effort and intentionality is the translation into execution and implementation (Rhodes and Rebar, 2017). Overall this group demonstrated how changed circumstance or lack of opportunity does not necessarily need to be a reason for not engaging in PA (Howlett *et al.*, 2019). As Dishman (Dishman, 2018) emphasizes, ideally regular healthy levels of PA needs to become part of one’s core identity.

This contrasts with those who mentioned loss of PA opportunities as a factor that negatively impacted PA. Lost opportunities included fewer opportunities for social interactions and a lack of access to usual facilities or structured events or organized sports. A lack of adequate spaces have been noted as barriers to PA by Alcántara-Porcuna *et al*. (Alcántara-Porcuna *et al.*, 2022), and previous research has also suggested that improved provision of exercise or sporting facilities can enhance PA uptake (Heath *et al.*, 2012; Halonen *et al.*, 2015). Our findings for the young people in this situation are consistent with the adults in Constandt *et al*. (Constandt *et al.*, 2020) study who were less likely to engage in PA when deprived of their usual activity. Active travel and incidental PA associated with study or work is a form of structured opportunity, however, during the pandemic lockdown, walking or cycling for many became a choice rather than a necessity. In the absence of other motivating factors, this incidental PA was not replaced. Overall this group of young people perhaps found it harder to close gaps between intent and execution, thus reinforcing the importance of structured opportunities that can be motivating ­influences for PA (Teixeira *et al.*, 2012; Rhodes and Dickau, 2013).

However, in accordance with Kemp *et al*. (Kemp *et al.*, 2020) and Eime *et al*. (Eime *et al.*, 2022), we caution that a sole focus on maximizing participation in organized or sports club environments might not be the most effective way of stimulating sustained engagement in PA for young people, especially in circumstances of change. Rather, there is a need to open up options to a broader range of PA opportunities including beyond structured settings (Jeanes *et al.*, 2021; O’Connor and Penney, 2021). There is also a need for targeting skills and capabilities to begin and sustain participation in non-organized PA, where a low level of physical competence and few resources are required. In this study, those who increased or maintained PA during the lockdown participated on their own terms and outside of a structured environment albeit with social support in some cases; they made the rules, set the times, chose the mode of engagement and set their own level of challenge. This highlights the importance of encouraging young people to continue to try new forms of activity until they find forms of PA that enhances motivation to engage.

Outside of structured or organized PA opportunities, other factors were seen to drive motivation for PA. These incorporated knowledge capabilities such as understanding health and well-being benefits associated with PA, as evidenced by those who mentioned PA as a way to get outside, shift mindset or lift mood, or maintain physical strength or fitness. It is clear that for some, lockdown afforded opportunities and intrinsic motivation they previously lacked and aligns with Eime *et al*. (Eime *et al.*, 2022) suggestion that the pandemic has supported some individuals to prioritize and begin a more active life. Varea and González-Calvo (Varea and González-Calvo, 2021) suggest that the pandemic circumstances may have alerted students and their teachers to possibilities and pathways for engaging in PA on their own, perhaps with the support of online forums, resonates here.

The events surrounding the COVID-19 pandemic may have influenced the prioritization of health. For some, engaging in PA was not a habit that continued absent of external influences, but social connections and encouragement or exhortation from family members or friends online made the difference, as also noted elsewhere (Heath *et al.*, 2012). In addition, findings draw attention to the importance of having or making time for PA, and the value of flexibility. If youth undertake long bus rides to get to their education institute there are time implications for reduced PA and this research perhaps reinforces the benefits of options to stay put and learn online. Similarly important was the ability to incorporate PA into a daily routine. These recommendations are in keeping with Van Dyck *et al*. (Van Dyck *et al.*, 2015) who identified self-efficacy, time management and increased knowledge of PA benefits as positively influencing non-organized PA behaviours in college and university students.

In sum, youth voices in this research have demonstrated both how easy and how hard it can be to maintain healthy levels of PA. Regarding opportunity, increasing access to activity-promoting infrastructure and environments is imperative (Jáuregui *et al.*, 2021). Alongside this and as discussed it will be important to work with adolescents themselves in areas of motivation and capability development to better understand what does and does not work for them, for example, in making the incidental more intentional. Remaining active over time requires the ability to adapt PA as circumstances change over the life course (Teare and Taks, 2021). This notion is of consequence during periods of transition in late adolescence and emerging adulthood when motives and opportunities for PA change as they shift to new environments (Dishman *et al.*, 2018).

Drawing attention to the need for more research and PA promotion programmes, van van Sluijs *et al*. (van Sluijs *et al.*, 2021) suggest targeting older adolescents as they are more likely to experience life transitions that contribute to declining levels of PA and associated health consequences. They argue that programmes should be codesigned with adolescents in school, drawing on the context-specific support that school settings can provide. Multisectoral collaborations between education and health have proven effective in supporting short and long-term learning and behaviour change targeted towards this goal (Bay et al., 2016, 2017). Further, van Sluijs *et al.* also point out that alternative strategies based on older adolescents’ needs and life circumstances must support the large proportion of young people not in school.

## CONCLUSION

Our research identifies possible leverage points and modifiable and non-modifiable factors impacting PA across individual, social and structural/environmental domains. Taken together, these findings have implications for how we might engage with and educate young people to develop their physical literacy during a life phase that is associated with increased autonomy and independence but also can present significant challenges and change. A key question is what young people themselves might find useful in this research to support them to plan for and prioritize PA. Further research could involve young people having opportunities to examine data for themselves or conduct their own small-scale investigation in this area, as learners are more likely to engage and take action if they produce and work with self-generated data. Analysis of COM-B constructs could support young people to identify for themselves some modifiable and non-modifiable factors and positive and negative influences contributing to PA. As Thorpe (Thorpe, 2021) recommends, creative strategies and initiatives prioritizing PA resilience should be devised by youth themselves. This study confirms that the type of PA matters less for strengthening PA participation than the expectation that PA needs to change to adapt to new circumstances over the life course, and the knowledge of modifiable factors that potentially support change. These insights are useful for other life transitions, such leaving the family home for independent living, where altered routines and circumstances might mean it is similarly difficult but nevertheless crucial for youth to sustain PA as part of who they are, what they do and how they live their life.

### Limitations

The questionnaire focussed on youth experiences of the lockdown across a range of categories. To keep length and response times to a minimum, a limited number of items were selected that focussed on PA. This meant we were not able to inquire in more detail into, for example, the length and frequency of PA, or why for some PA might have been limited prior to the pandemic. The findings do provide a basis for understanding changes in PA behaviour over the lockdown. The large sample size supports further in-depth analysis and discussion pursuing the significance of associations between various demographic factors against specific changes in types and levels of PA participation, which is a focus for future work. Future research could use focus group approaches to investigate the type and impact of varying motivations and capabilities related to PA.
